# Supplementary material for: Molecular recognition by multiple metal coordination inside wavy-stacked macrocycles
Source: Nat Commun. 2017 Jul 25;8:129. doi: 10.1038/s41467-017-00076-8 (PMC5527018; doi:10.1038/s41467-017-00076-8)
Supplement: Supplementary file 1 — Supplementary Information [file 41467_2017_76_MOESM1_ESM.pdf]

File Name: Supplementary Information
Description: Supplementary Figures, Supplementary Table, Supplementary Methods and Supplementary References

File Name: Peer Review File
Description:

Supplementary Methods

Silica gel for column chromatography was purchased from Kanto Chemical Co. Inc. (Silica Gel 60 N (spherical, 63–210 mm or 40–50 μm). Dry DMF was purified by Nikko Hansen Ultimate Solvent System 3S-TCN 1. See also Methods section in the main text for general information.

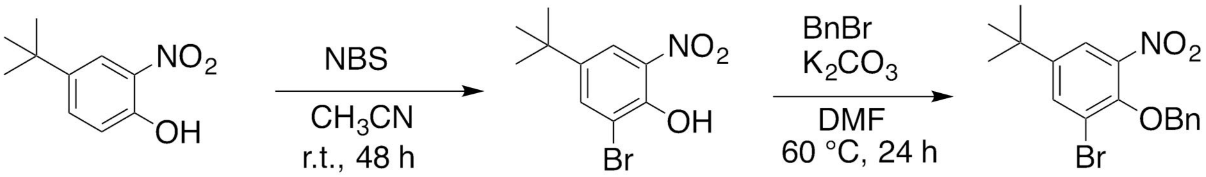

5 6 (54%) 7 (67%)

Supplementary Figure 1. Syntheses of **6** and **7**

Synthesis of **6**

A solution of **5** [1] (1.01 g, 5.20 mmol, 1.0 eq.) and NBS (0.94 g, 5.31 mmol, 1.1 eq.) in dry CH_3CN (30 mL) was stirred for 48 h at room temperature under Ar atmosphere. The reaction mixture was quenched with sat. $NaHSO_3$ aq. (40 mL) and extracted with $CHCl_3$ (100 mL × 2). The organic layer was washed with H_2O (50 mL), dried over $MgSO_4$, filtered, and concentrated in vacuo. The residue was purified by column chromatography on silica gel ($CHCl_3$) to give **6** as a yellow solid (770.9 mg, 2.82 mmol, 54%).

Melting point: 77.0–78.0 °C;

^{1}H NMR (400 MHz, $CDCl_3$): δ 10.99 (d, J = 0.5 Hz, 1H), 8.07 (d, J = 2.4 Hz, 1H), 7.90 (dd, J = 2.4, 0.5 Hz, 1H), 1.33 (s, 9H);

^{13}C NMR (101 MHz, $CDCl_3$): δ 150.0, 144.2, 138.7, 133.7, 120.7, 112.9, 34.5, 31.0;

Elemental analysis: calcd for $C_{10}H_{12}BrNO_3$ (**6**); H, 4.41; C, 43.82; N, 5.11, found H, 4.42; C, 43.91; N, 5.12.

Synthesis of **7**

A mixture of **6** (100.6 mg, 0.366 mmol, 1.0 eq.), K_2CO_3 (57.1 mg, 0.413 mmol, 1.1eq.) and benzyl bromide (44 μL, 0.366 mmol, 1.0 eq.) in dry DMF (1.0 mL) was stirred for 24 h at 60 °C under Ar atmosphere. To the reaction mixture was added H_2O (20 mL) and extracted with Et_2O (20 mL × 4). The organic layer was washed with brine (50 mL), dried over $MgSO_4$, filtered, and concentrated in vacuo. The residue was purified by column chromatography on silica gel (hexane/CH_2Cl_2 = 3/1) to give **7** as a pale yellow oil (89.4 mg, 0.246 mmol, 67%).

^{1}H NMR (400 MHz, $CDCl_3$): δ 7.81 (d, J = 2.4 Hz, 1H), 7.76 (d, J = 2.4 Hz, 1H), 7.57–7.54 (m, 2H), 7.43–7.37 (m, 3H), 5.16 (s, 2H), 1.34 (s, 9H);

^{13}C NMR (101 MHz, $CDCl_3$): δ 149.3, 146.7, 145.1, 135.7, 135.1, 128.7, 128.6, 128.5, 131.5, 119.7, 76.4, 34.9, 30.9;

HRMS (ESI): m/z calcd for ([**7**·Na^+]): 386.0368; found: 386.0353.

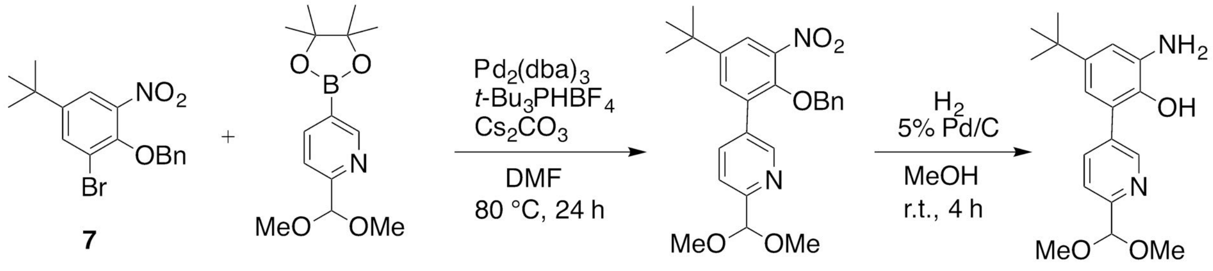

Supplementary Figure 2. Syntheses of **9** and **2**

Synthesis of **9**

A mixture of **7** (1.02 g, 2.82 mmol, 1.0 eq.), **8**[2] (1.34 g, 4.79 mmol, 1.7 eq.), $Pd_2(dba)_3$ (94.7 mg, 91.5 μmol, 0.03 eq.), *t*-Bu_3PHBF_4 (99.5 mg, 338 μmol, 0.12 eq.), and Cs_2CO_3 (2.87 g, 8.81 mmol, 3.1eq.) in dry DMF (10 mL) was stirred for 24 h at 80 °C under Ar atmosphere. To the reaction mixture was added H_2O (50 mL) and extracted with Et_2O (100 mL × 3). The organic layer was washed brine (50 mL), dried over $MgSO_4$, filtered, and concentrated in vacuo. The residue was purified by column chromatography on silica gel (EtOAc/hexane = 1/4) to give **9** as a yellow oil (1.07 g, 2.45 mmol, 87%).

^{1}H NMR (400 MHz, $CDCl_3$): δ 8.78 (dd, *J* = 2.2, 0.5 Hz, 1H), 7.95 (dd, *J* = 8.1, 2.2 Hz, 1H), 7.84 (d, *J* = 2.5 Hz, 1H), 7.61 (dd, *J* = 8.1, 0.5 Hz, 1H), 7.56 (d, *J* = 2.5 Hz, 1H), 7.29–7.22 (m, 3H), 7.07–7.05 (m, 2H), 5.46 (s, 1H), 4.64 (s, 2H), 3.47 (s, 6H), 1.38 (s, 9H);

^{13}C NMR (101 MHz, $CDCl_3$): δ 156.9, 149.0, 148.4, 146.9, 144.9, 137.4, 135.2, 134.1, 132.6, 132.2, 128.6, 128.5, 128.4, 121.9, 120.8, 103.7, 76.7, 53.7, 34.9, 31.1;

HRMS (ESI): *m*/*z* calcd for ([**9**·K^+]): 475.1635; found: 475.1668.

Synthesis of **2**

A mixture of **9** (1.12 g, 2.58 mmol, 1.0 eq.) and 5% Pd/C (550 mg, 5.17 mmol, 2.0 eq.) in dry MeOH (16 mL) was stirred for 4 h at room temperature under H_2. The reaction mixture was filtered, and the filtrate was concentrated in vacuo. To the residue was added diisopropyl ether and concentrated in vacuo to give **2**·0.3H_2O as an orange solid (756 mg, 2.35 mmol, 91%).

Melting point: 128.5–129.5 °C;

^{1}H NMR (400 MHz, $CDCl_3$): δ 8.75 (d, *J* = 1.9 Hz, 1H), 7.91 (dd, *J* = 8.0, 1.9 Hz, 1H), 7.63 (d, *J* = 8.0 Hz, 1H), 6.87 (d, *J* = 2.3 Hz, 1H), 6.69 (d, *J* = 2.3 Hz, 1H), 5.41 (s, 1H), 3.45 (s, 6H), 1.29 (s, 9H);

^{13}C NMR (101 MHz, $CDCl_3$): δ 156.2, 149.3, 144.4, 139.3, 137.4, 134.5, 133.9, 124.1, 121.2, 117.5, 114.8, 104.1, 54.0, 34.2, 31.5;

Elemental analysis: calcd for $C_{18}H_{24.6}N_2O_{3.3}$ (**2**·0.3H_2O); H, 7.71; C, 67.18; N, 8.71. found H, 7.66; C, 66.91; N, 8.54.

HRMS (ESI): *m*/*z* calcd for ([**2**·Na^+]): 339.1685; found: 339.1694.

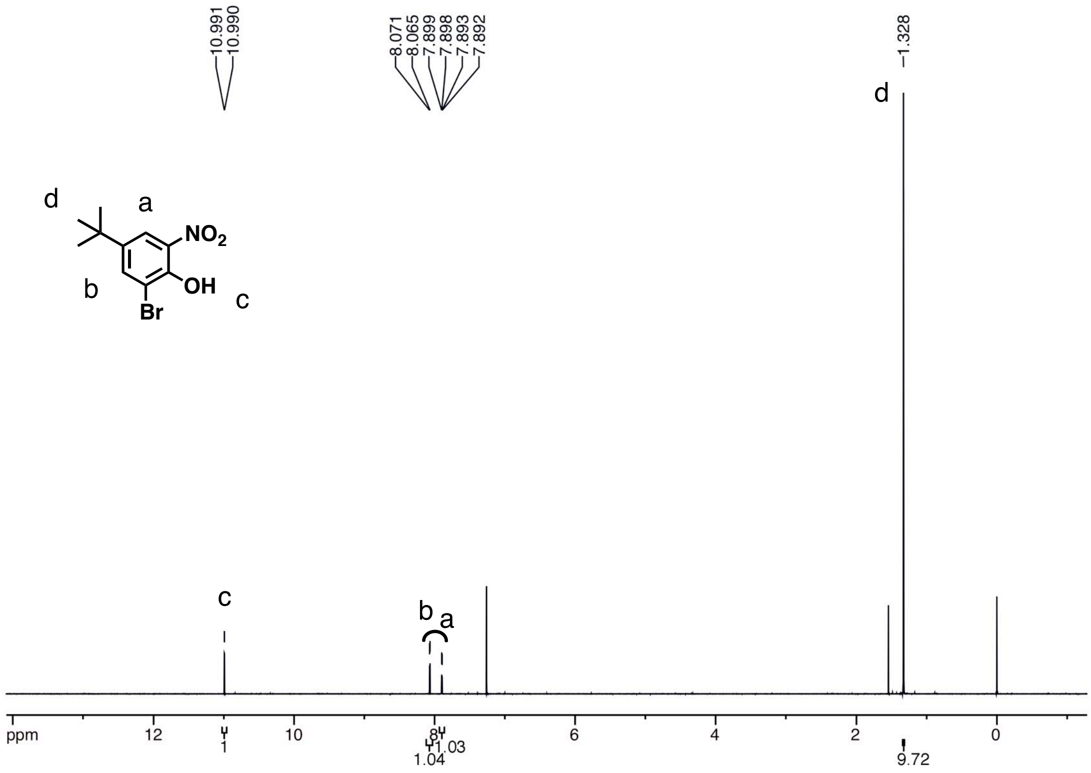

Supplementary Figure 3. ^{1}H NMR spectrum of **6** (400 MHz, $CDCl_3$).

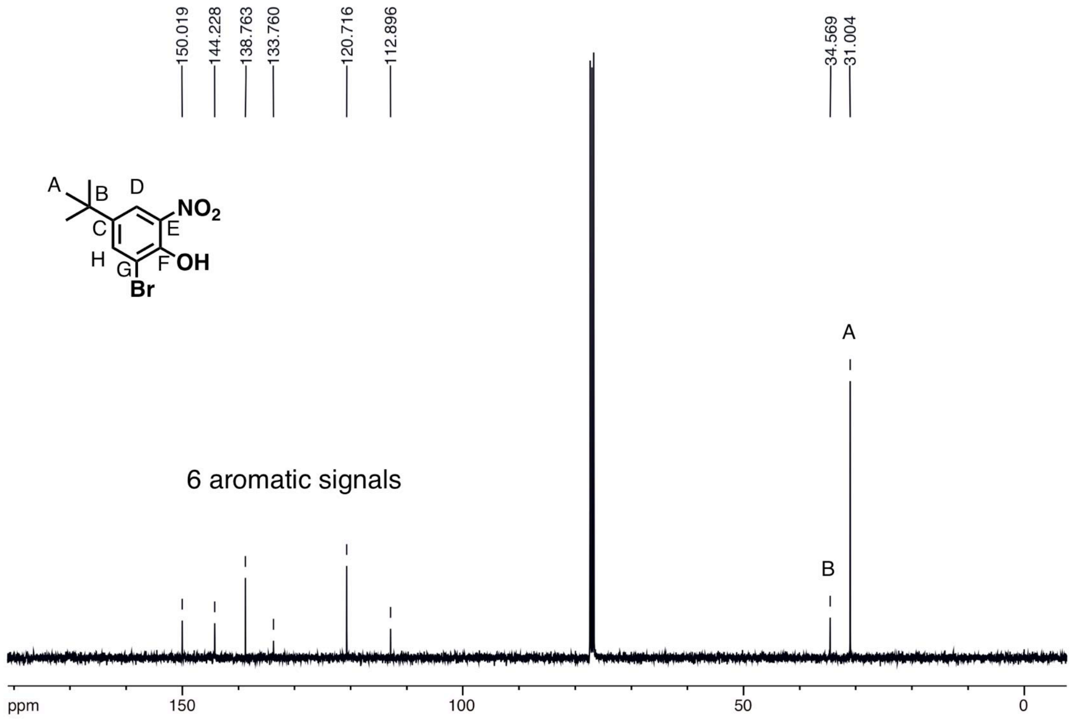

Supplementary Figure 4. ^{13}C NMR spectrum of **6** (101 MHz, $CDCl_3$).

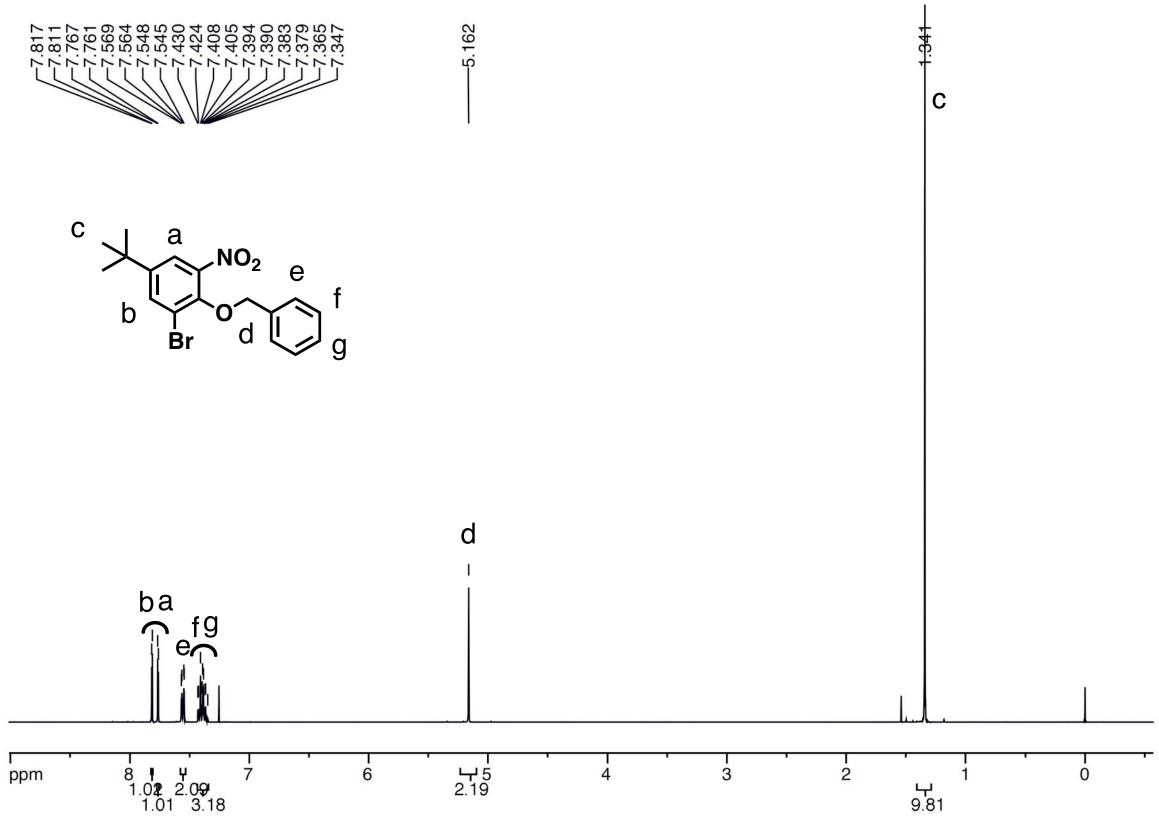

Supplementary Figure 5. ^{1}H NMR spectrum of **7** (400 MHz, $CDCl_3$).

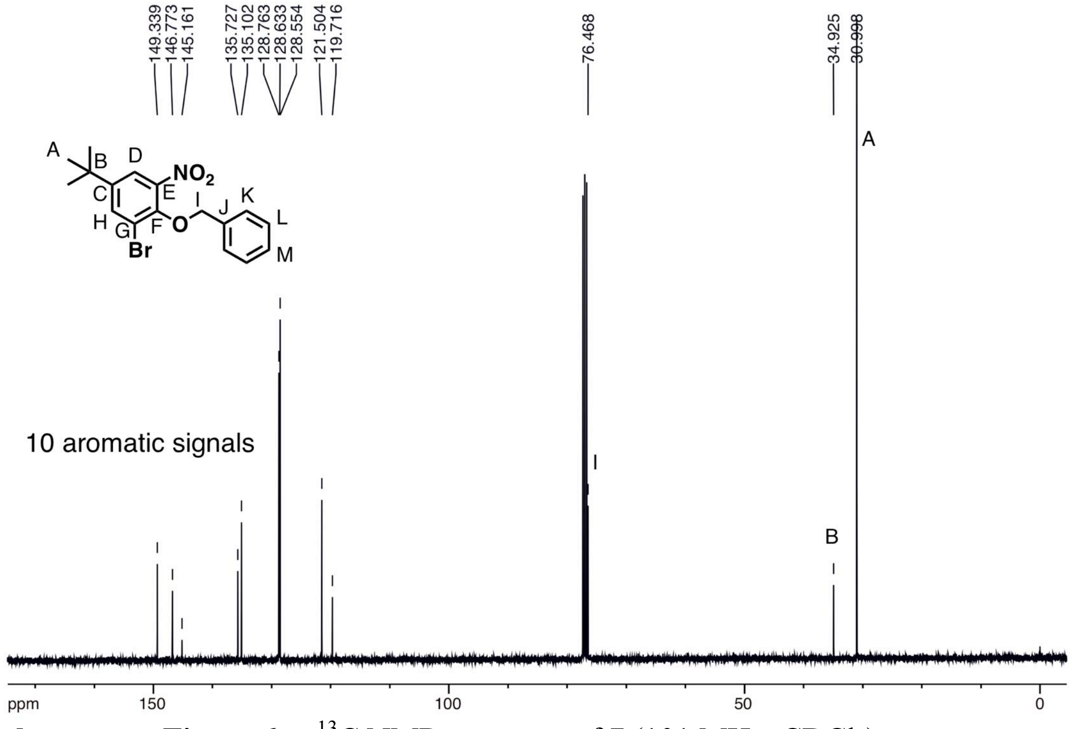

Supplementary Figure 6. ^{13}C NMR spectrum of **7** (101 MHz, $CDCl_3$).

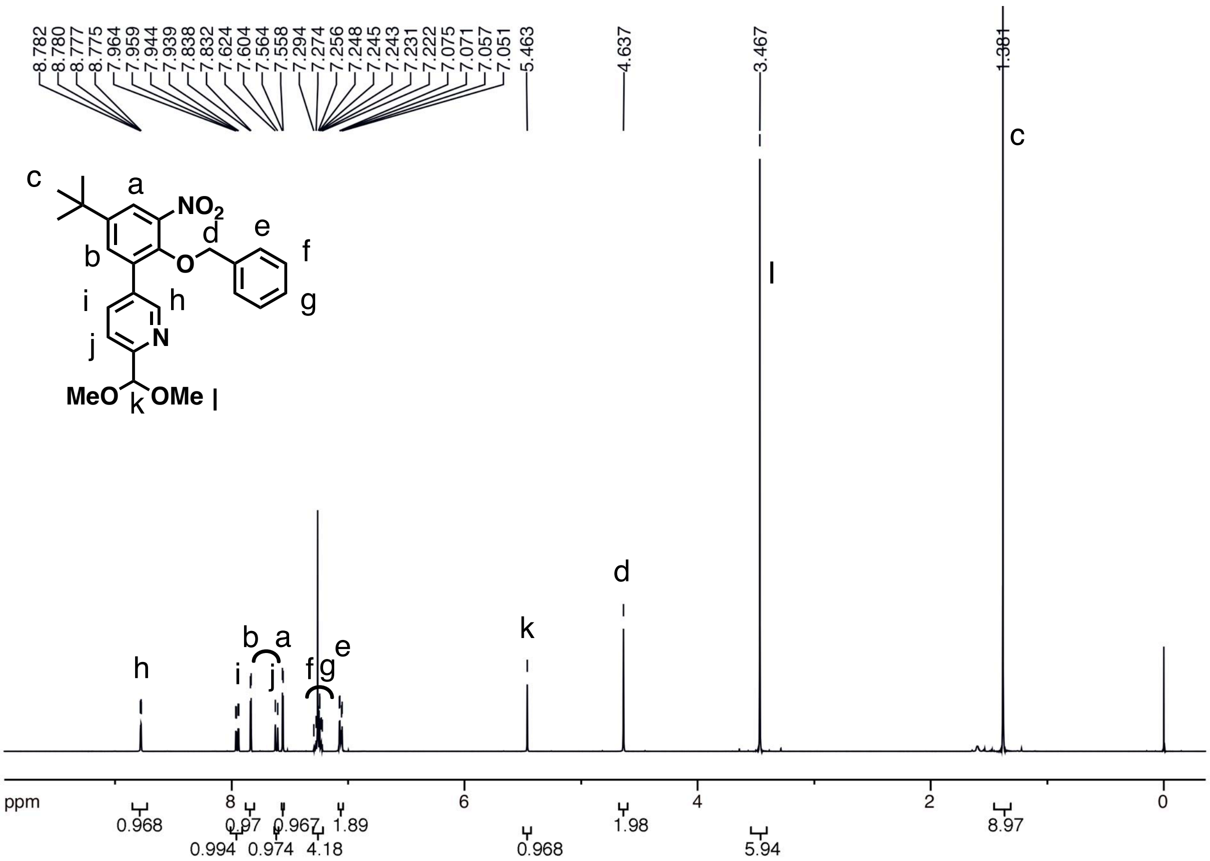

Supplementary Figure 7. ^{1}H NMR spectrum of **9** (400 MHz, $CDCl_3$).

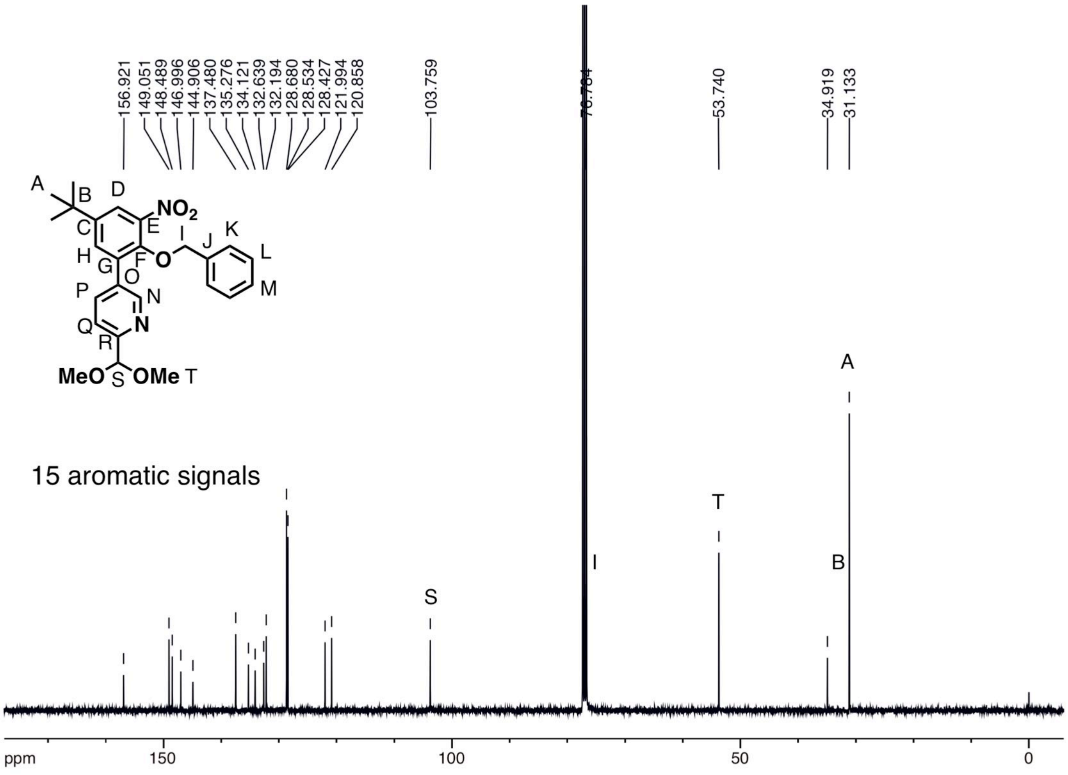

Supplementary Figure 8. ^{13}C NMR spectrum of **9** (101 MHz, $CDCl_3$).

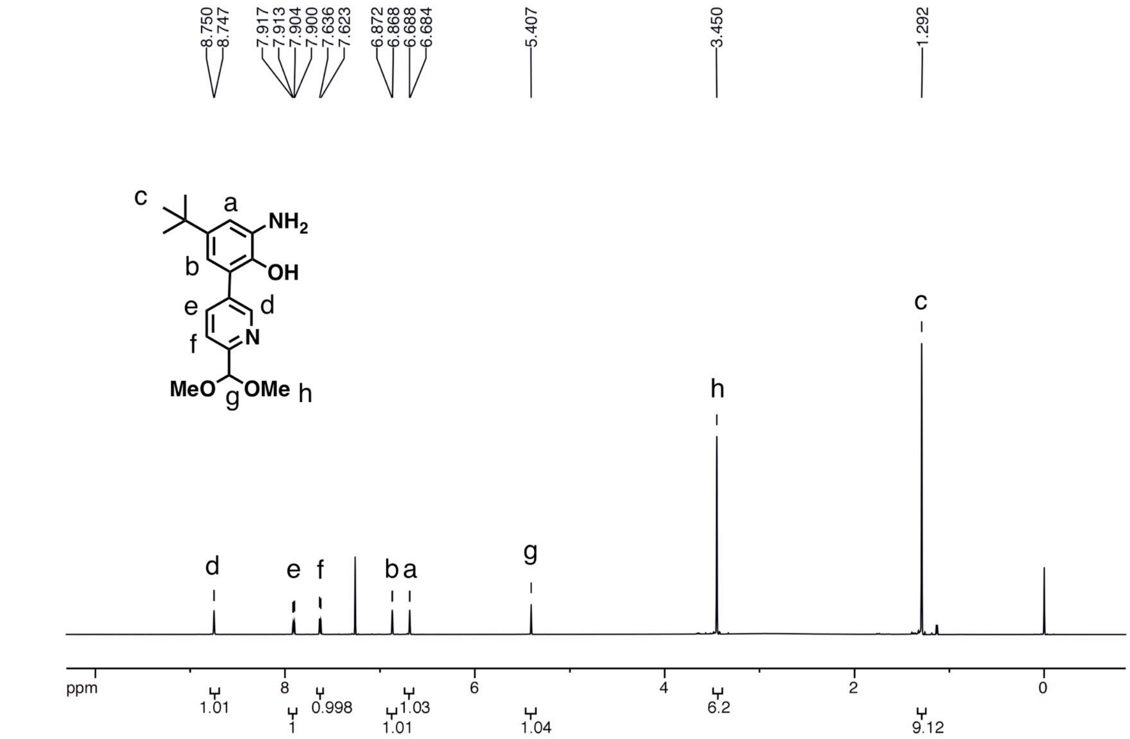

Supplementary Figure 9. ^{1}H NMR spectrum of **2** (400 MHz, $CDCl_3$).

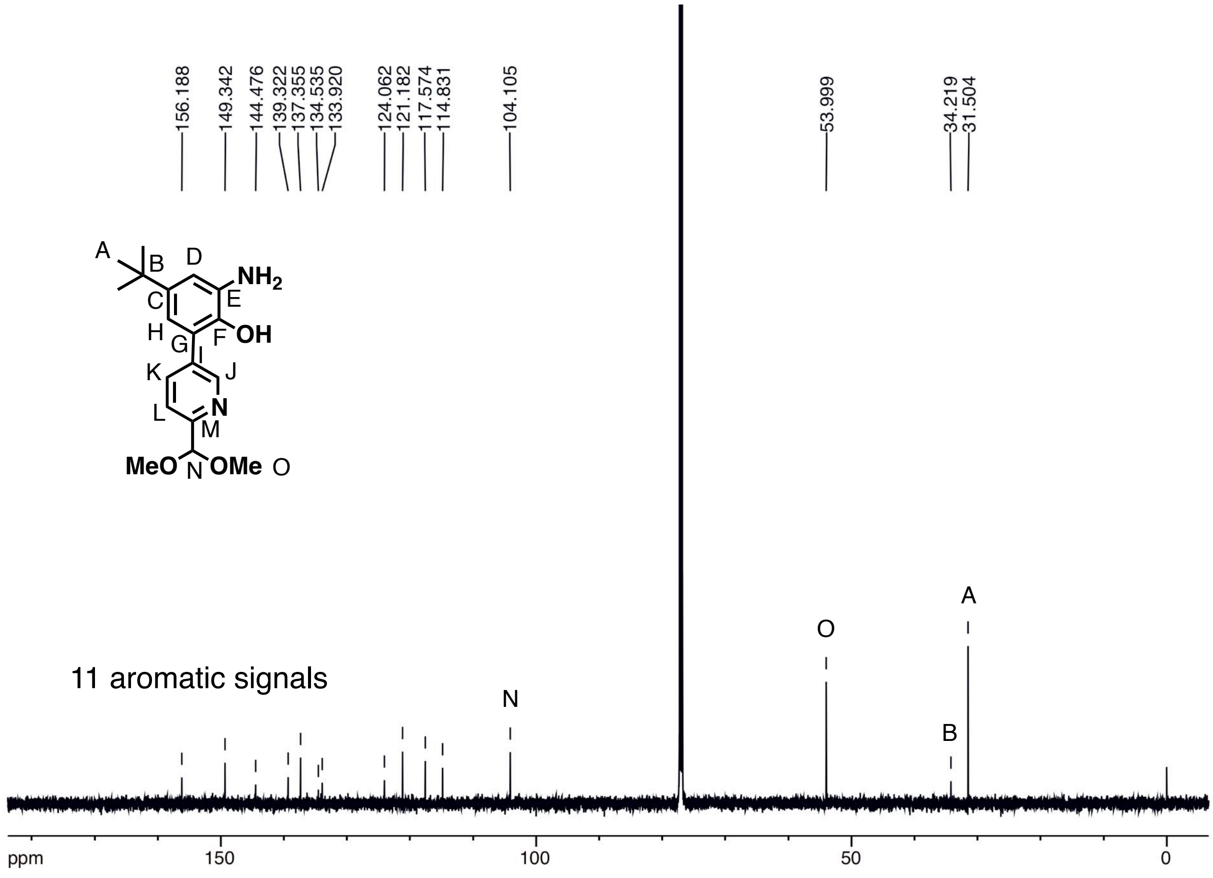

Supplementary Figure 10. ^{13}C NMR spectrum of **2** (101 MHz, $CDCl_3$).

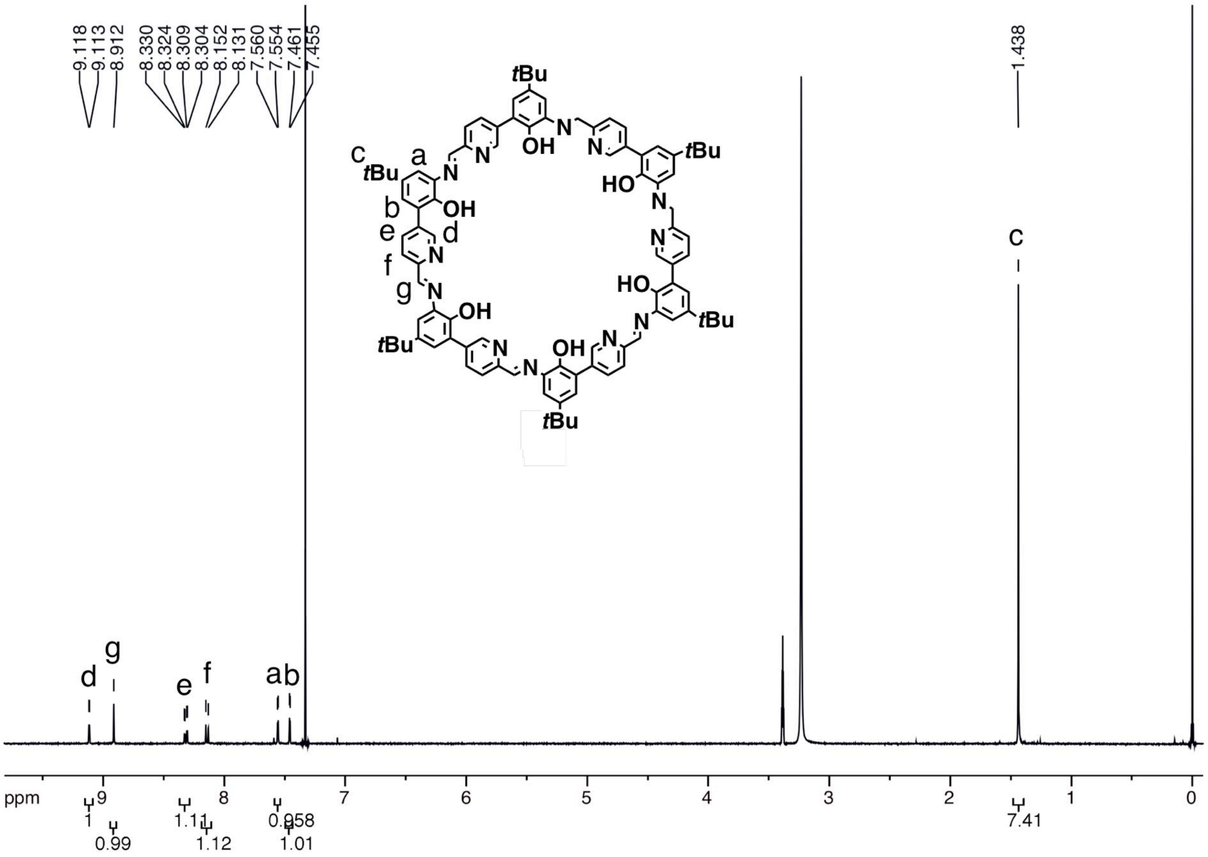

Supplementary Figure 11. ^{1}H NMR spectrum of H_6**1** (400 MHz, $CDCl_3$/CD_3OD = 10:1).

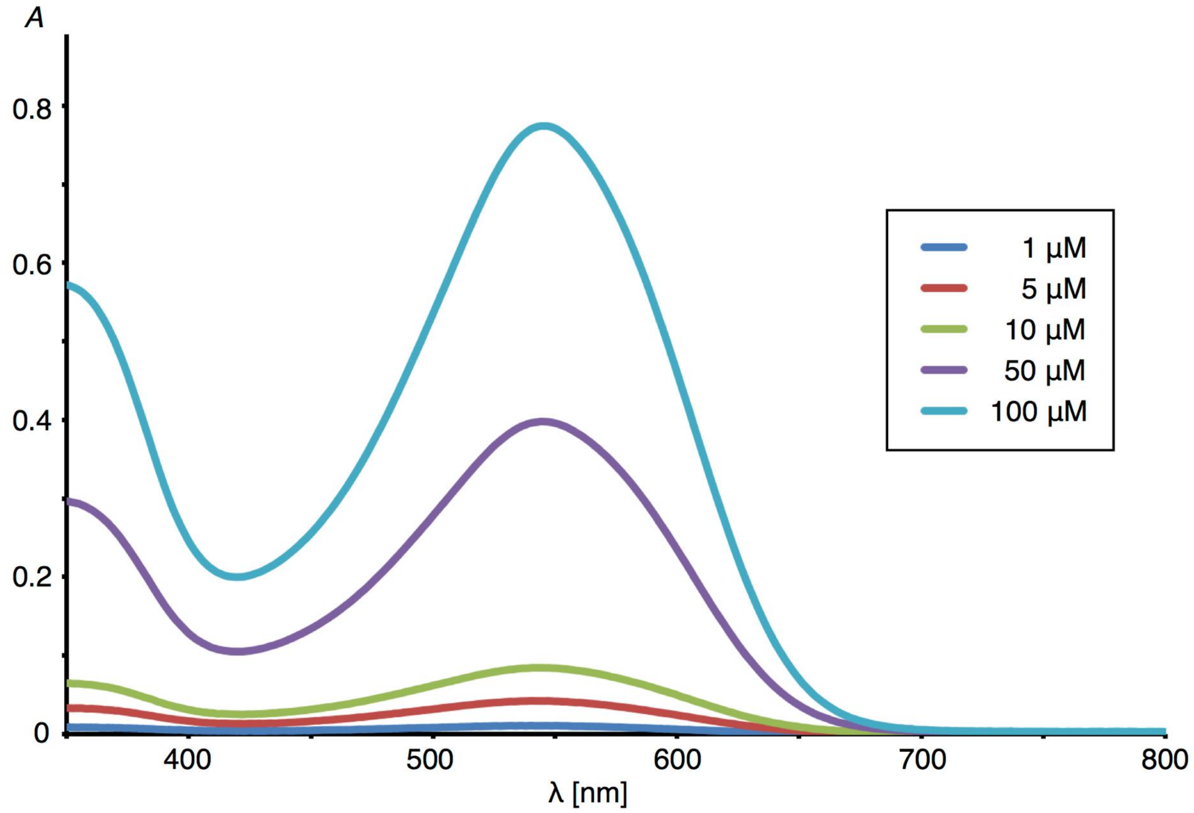

Supplementary Figure 12. UV-Vis absorption spectra of Zn-hexapap [**1**$Zn_6(acac)_6$] in various concentrations ($CHCl_3/CH_3OH$ = 10/1 (v/v), 298 K, *l* = 1.0 mm).

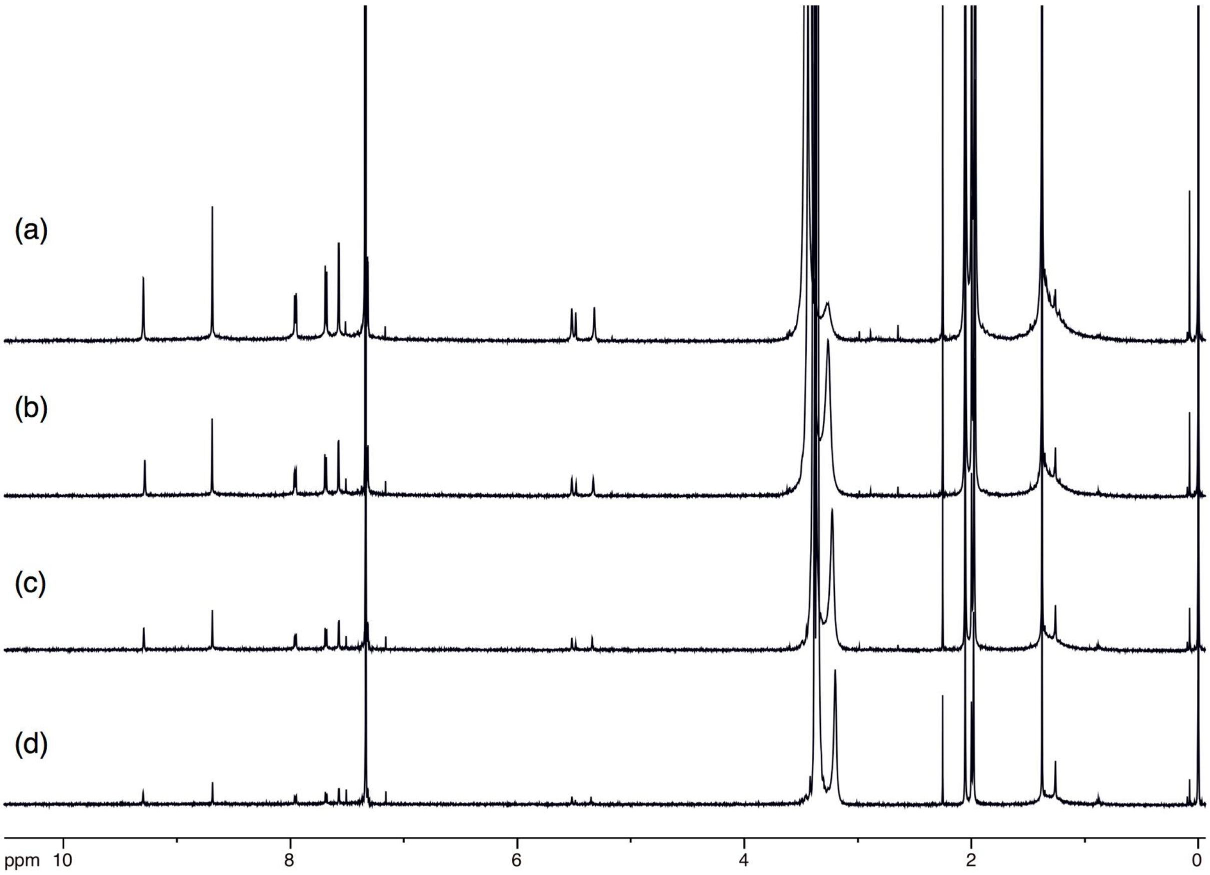

Supplementary Figure 13. ^{1}H NMR spectra of Zn-hexapap [**1**$Zn_6(acac)_6$] in various concentrations (600 MHz, $CDCl_3/CD_3OD$ = 10/1 (v/v), 298 K). (a) 1.2 mM. (b) 0.6 mM. (c) 0.3 mM. (d) 0.15 mM.

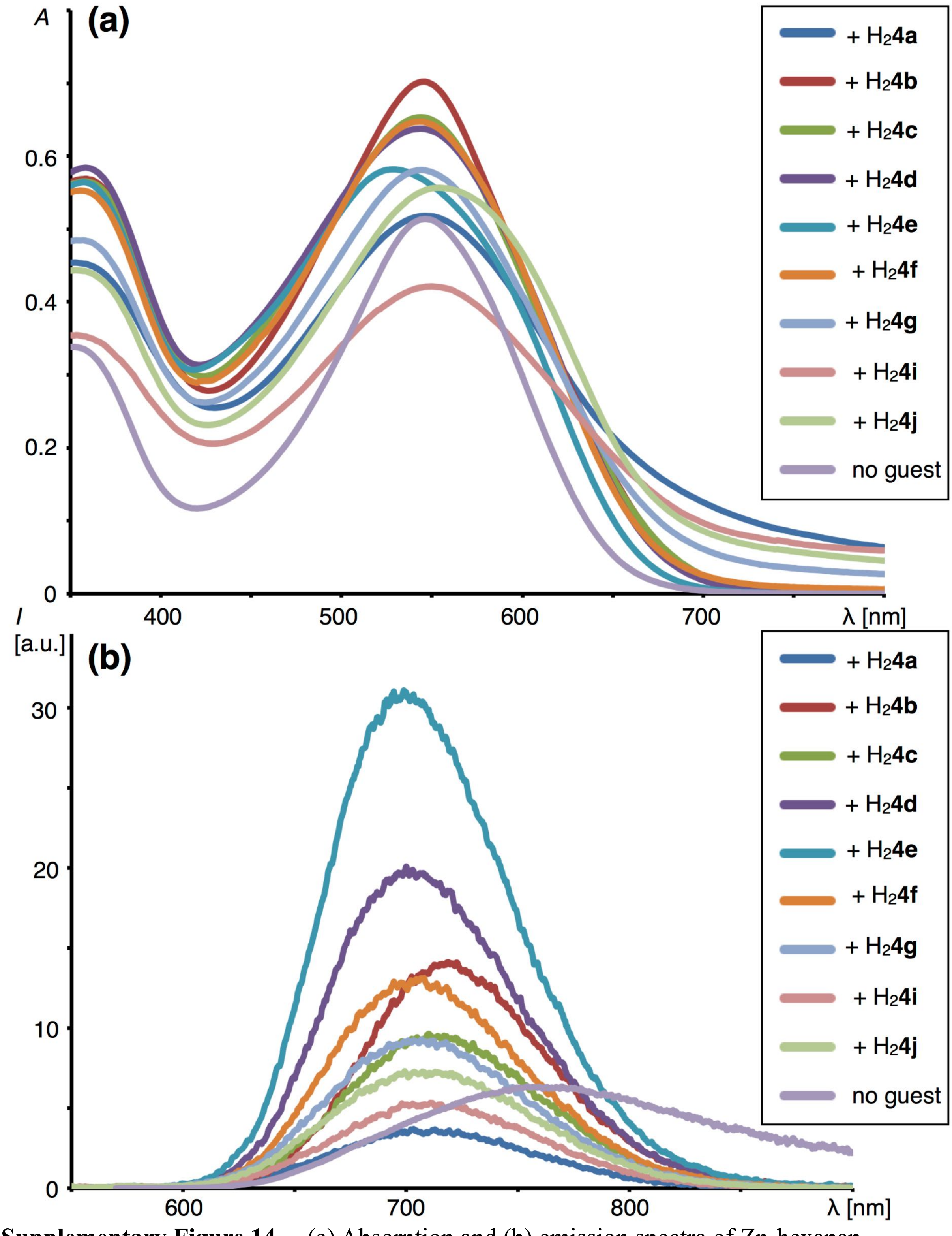

Supplementary Figure 14. (a) Absorption and (b) emission spectra of Zn-hexapap [**1**$Zn_6(acac)_2$] upon binding of a series of dicarboxylic acids H_2**4a**–H_2**4i** (10 μM (for the spectrum of the sample with no guest, 5 μM), $CHCl_3/CH_3OH$ = 10/1 (v/v), 298 K, l = 1.0 cm, λ_{ex} = 490 nm for (b)). Each sample was prepared by diluting the corresponding NMR sample (Fig. 2a–2i).

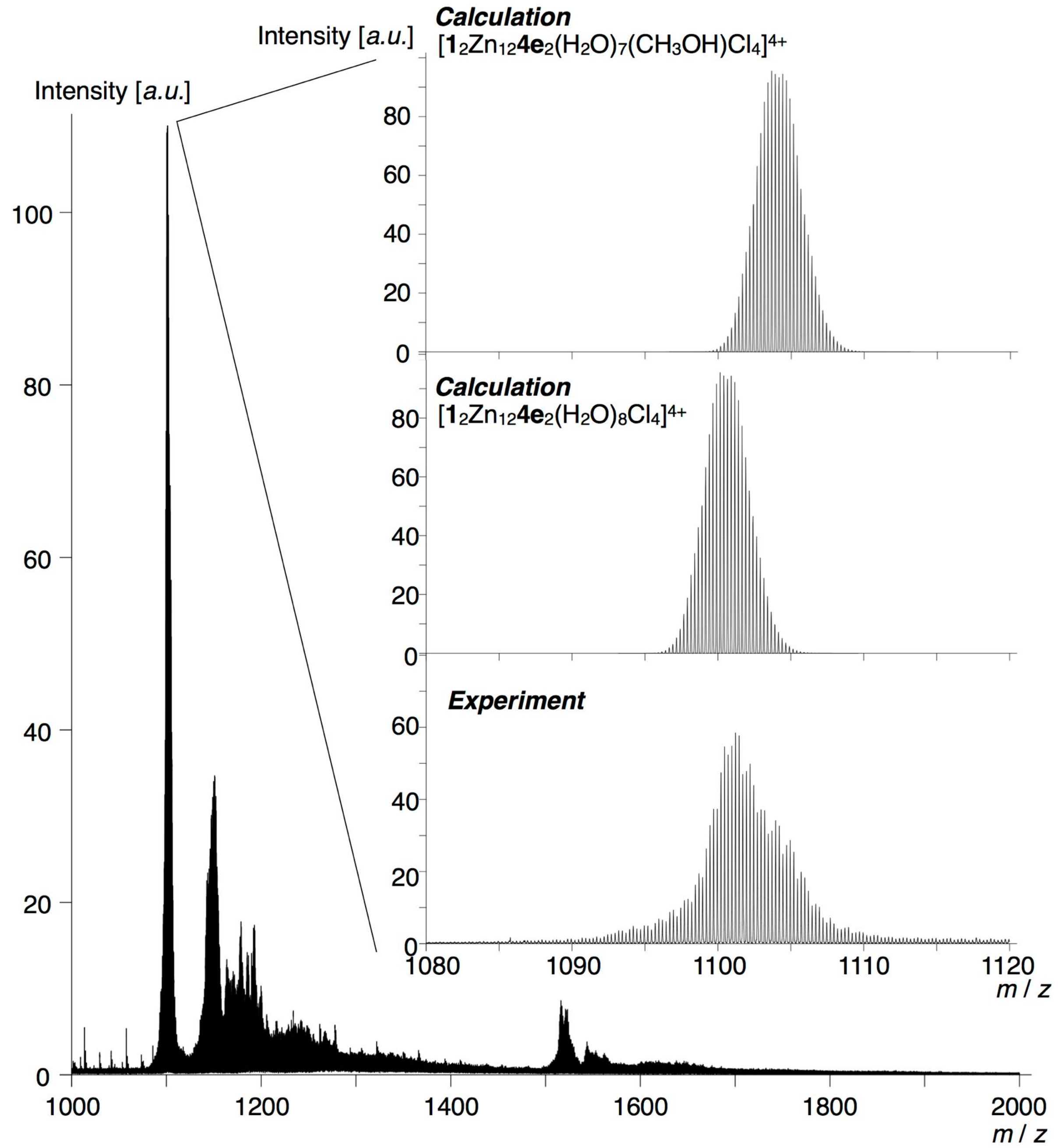

Supplementary Figure 15. ESI TOF mass spectrum of [$\mathbf{1}_2$Zn$_{12}$$\mathbf{4e}_2X_n$] (Solvent: CH_3OH). The strongest peak was assigned to be the overlapped signals of [$\mathbf{1}_2$Zn$_{12}$$\mathbf{4e}_2$($H_2O$)$_8Cl_4$]$^{4+}$ and [$\mathbf{1}_2$Zn$_{12}$$\mathbf{4e}_2$($H_2O$)$_7$($CH_3OH$)Cl$_4$]$^{4+}$.

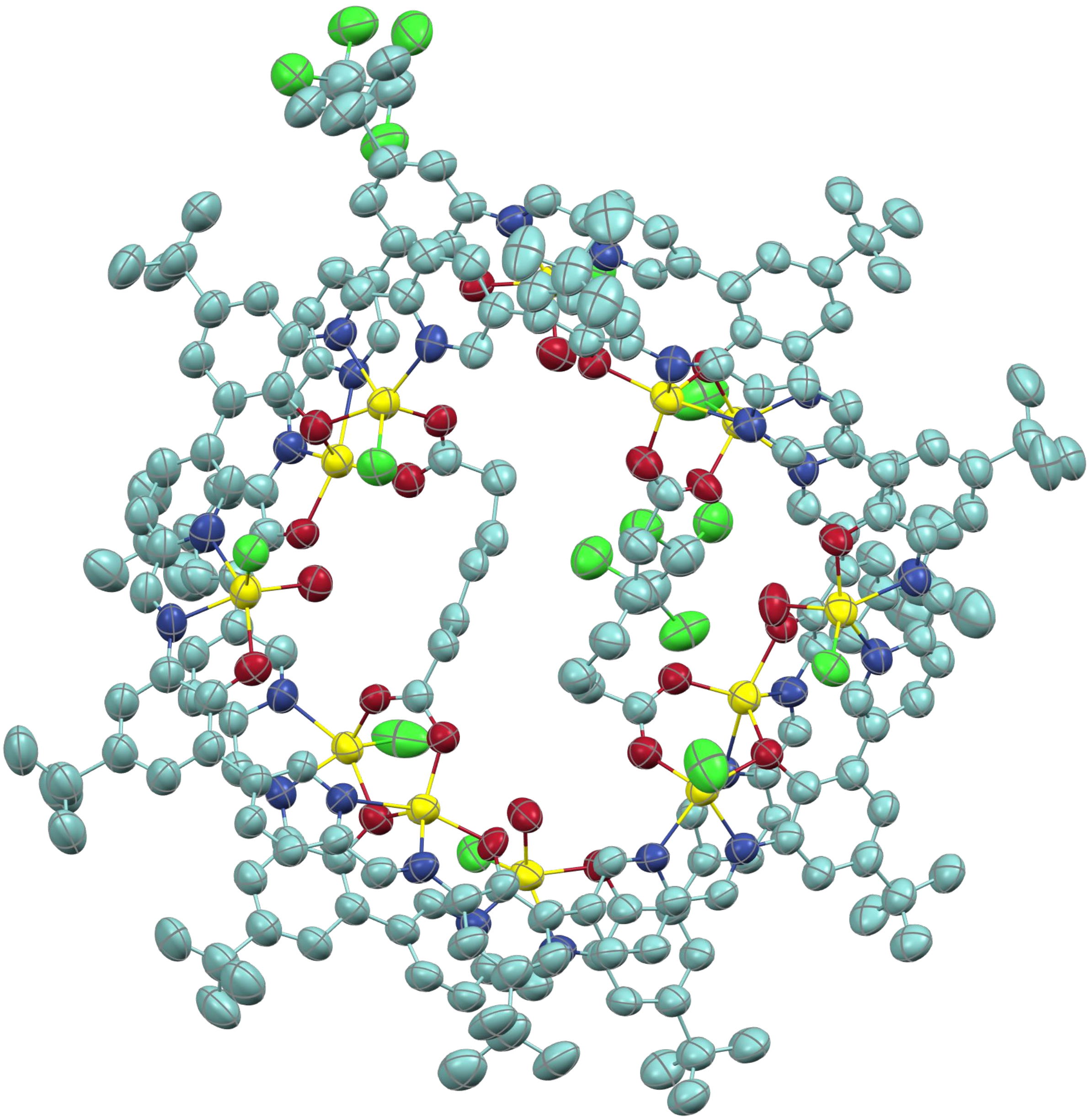

Supplementary Figure 16. The molecular structure of [$\mathbf{1}_2Zn_{12}\mathbf{4e}_2(H_2O)_4Cl_8$]·1.5($CH_2Cl_4$) determined by X-ray diffraction analysis. An ellipsoidal model (30% probability). Hydrogen atoms were omitted for clarity. One disorder pattern of $\mathbf{4e}^{2-}$ is shown. C, light green; N, blue; O, red; B, yellow; Cl, green.

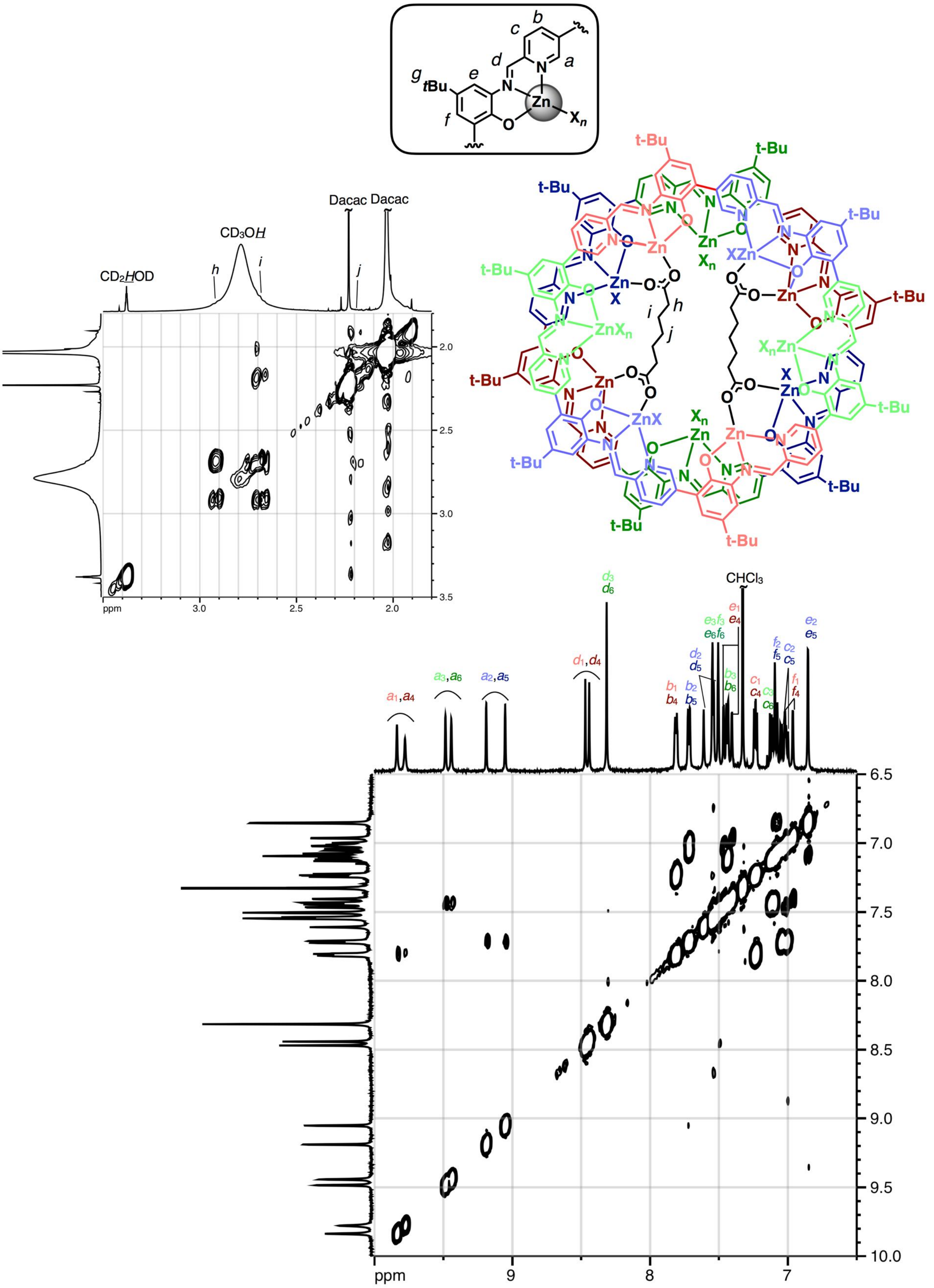

Supplementary Figure 17. ^1H–^1H COSY NMR spectrum of [$\mathbf{1}_2Zn_{12}\mathbf{4e}_2X_n$] (X = labile coordinating ligand) (600 MHz, $CDCl_3/CD_3OD$ = 10:1, 323 K).

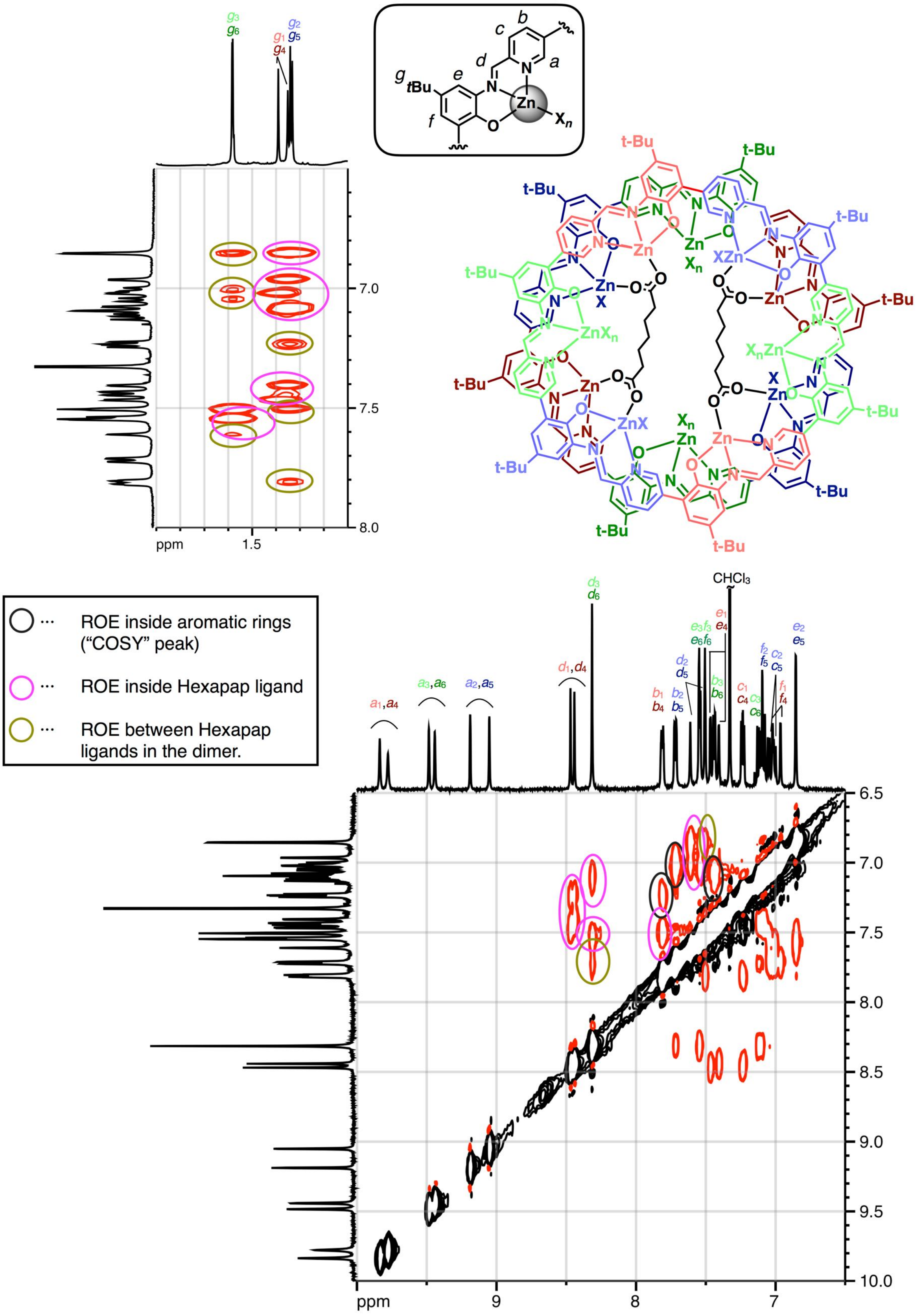

Supplementary Figure 18. ^{1}H–^{1}H ROESY NMR spectrum of [$\mathbf{1}_2Zn_{12}\mathbf{4e}_2X_n$] (X = labile coordinating ligand) (600 MHz, $CDCl_3/CD_3OD$ = 10:1, 323 K). Negative values are depicted in red in the spectrum.

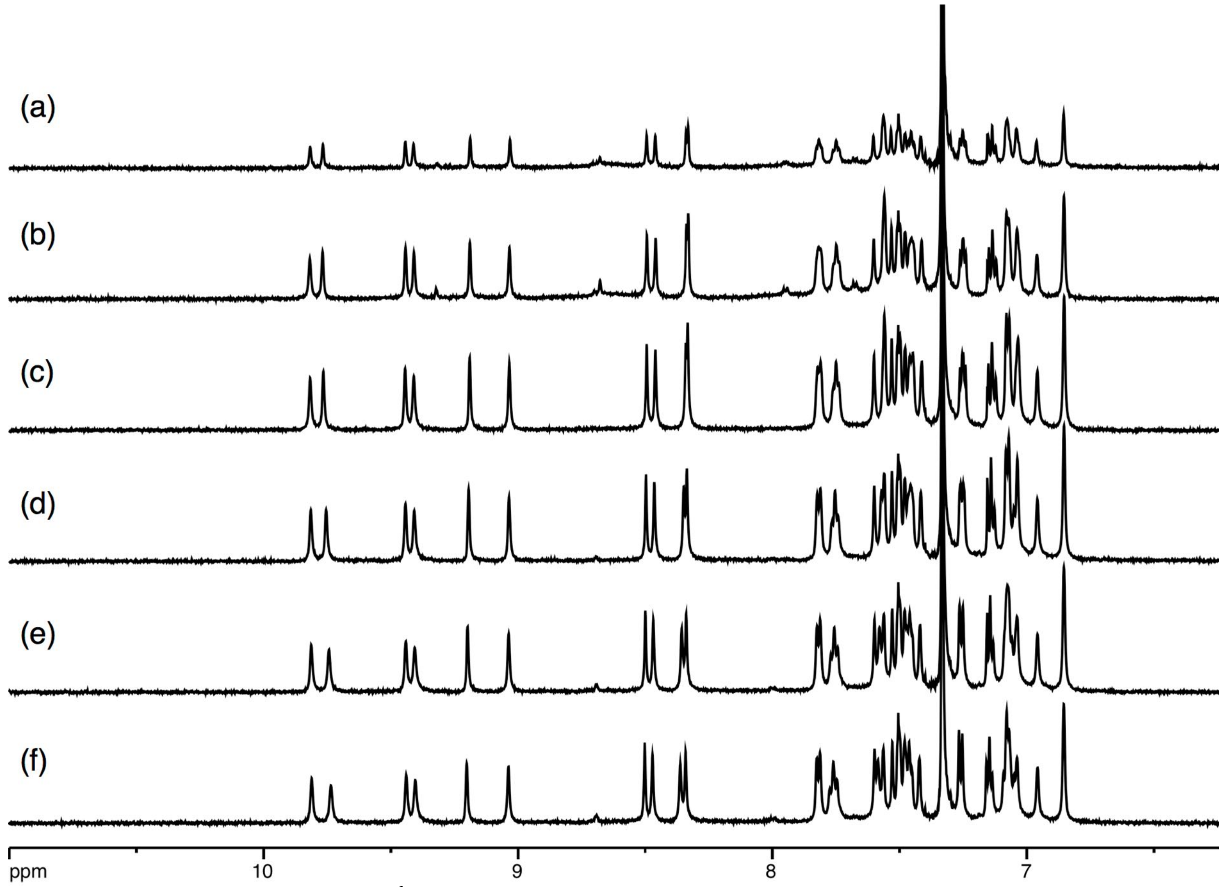

Supplementary Figure 19. ^{1}H NMR spectra of [$\mathbf{1}_2Zn_{12}\mathbf{4e}_2X_n$] with different amounts of pimelic acid $H_2\mathbf{4e}$ (400 MHz, $CDCl_3/CD_3OD$ = 10:1 (v/v), 298 K). (a) $H_2\mathbf{4e}$ 1 eq (/[$\mathbf{1}Zn_6X_n$]). (b) 2 eq. (c) 3 eq. (d) 4 eq. (e) 5 eq. (f) 6 eq.

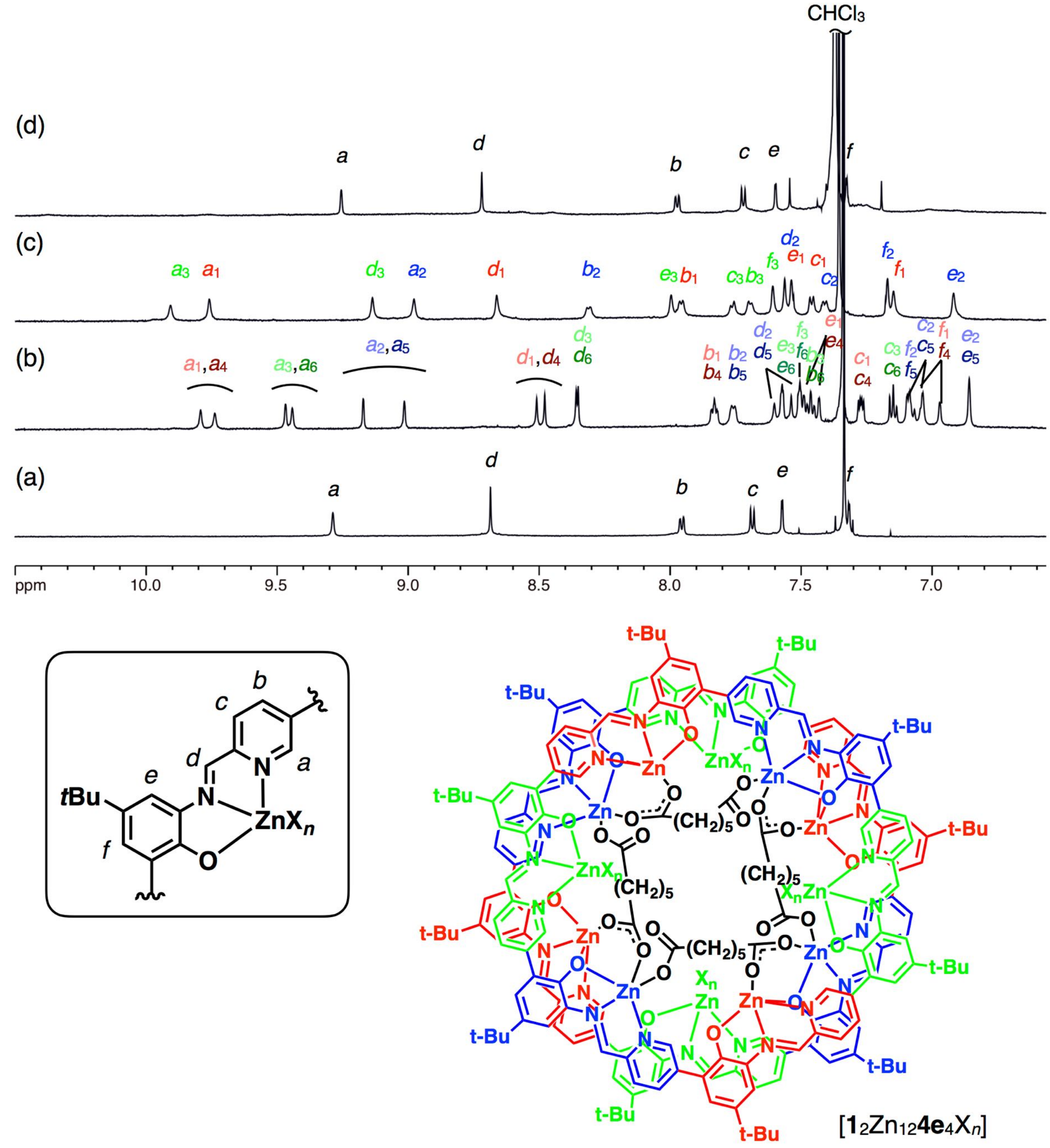

Supplementary Figure 20. Control of binding of pimelic acid H_4**4e** by acid/base stimuli. (^{1}H NMR, 600 MHz, $CDCl_3/CD_3OD$ = 10:1, 298 K). (a) Zn-hexapap [**1**$Zn_6(acac)_6$]. (b) Dimer of Zn-hexapap in bimolecular recognition mode, [$\mathbf{1}_2Zn_{12}\mathbf{4e}_2X_n$] ((a) + H_4**4e** (2 eq /[$\mathbf{1}^{6-}$])). See Figure 2k in the main text for the coloring of [$\mathbf{1}_2Zn_{12}\mathbf{4e}_2X_n$]. (c) Dimer of Zn-hexapap in tetramolecular recognition mode, [$\mathbf{1}_2Zn_{12}\mathbf{4e}_4X_n$] ((b) + CF_3SO_3H (3 eq /[$\mathbf{1}^{6-}$])). (d) Guest-free Zn-hexapap [**1**Zn_6X_n] ((c) + Me_4NOH (14 eq /[$\mathbf{1}^{6-}$])).

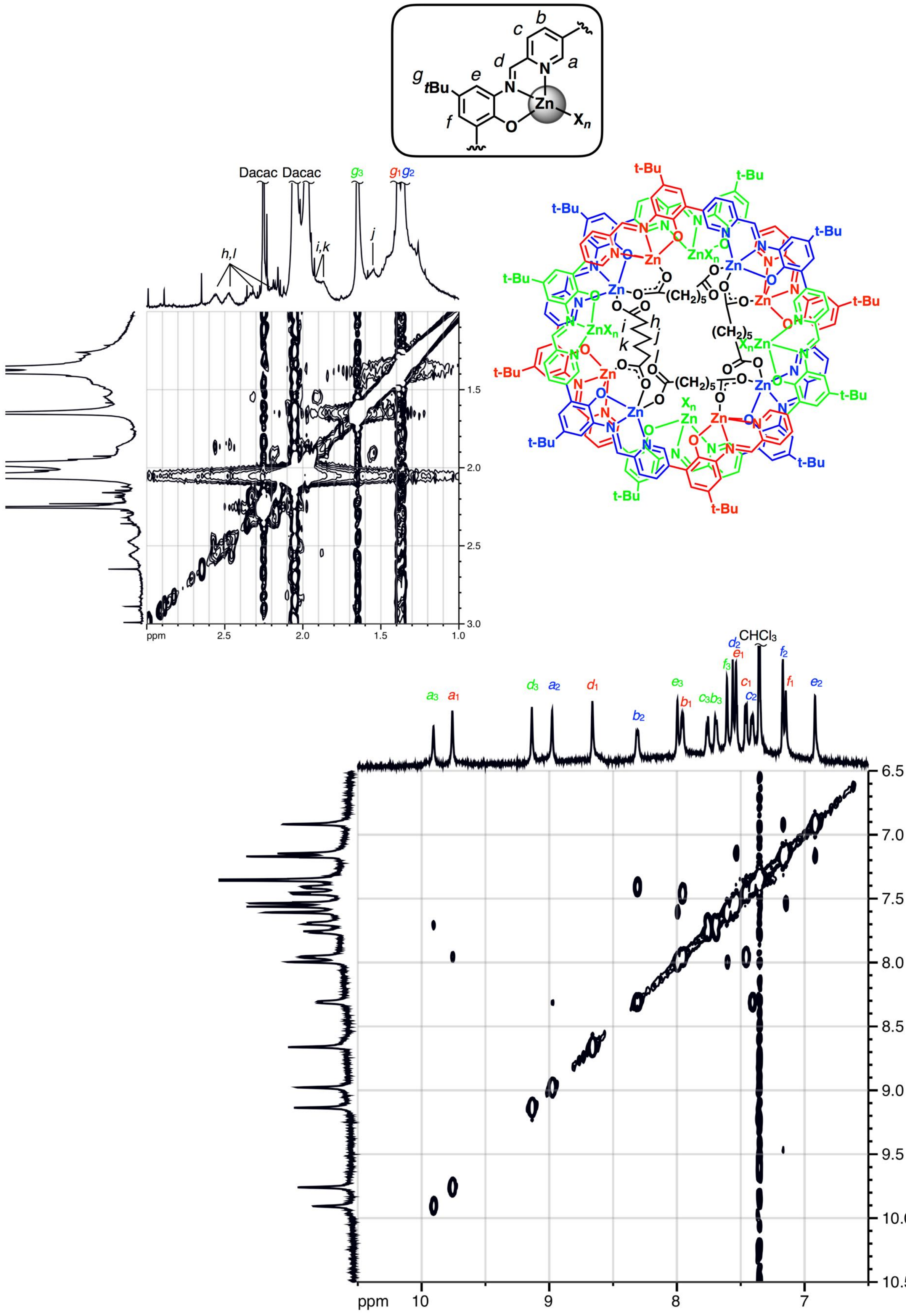

Supplementary Figure 21. ^{1}H–^{1}H COSY NMR spectrum of [$\mathbf{1}_2Zn_{12}\mathbf{4e}_4X_n$] (X = labile coordinating ligand) (600 MHz, $CDCl_3/CD_3OD$ = 10:1, 298 K).

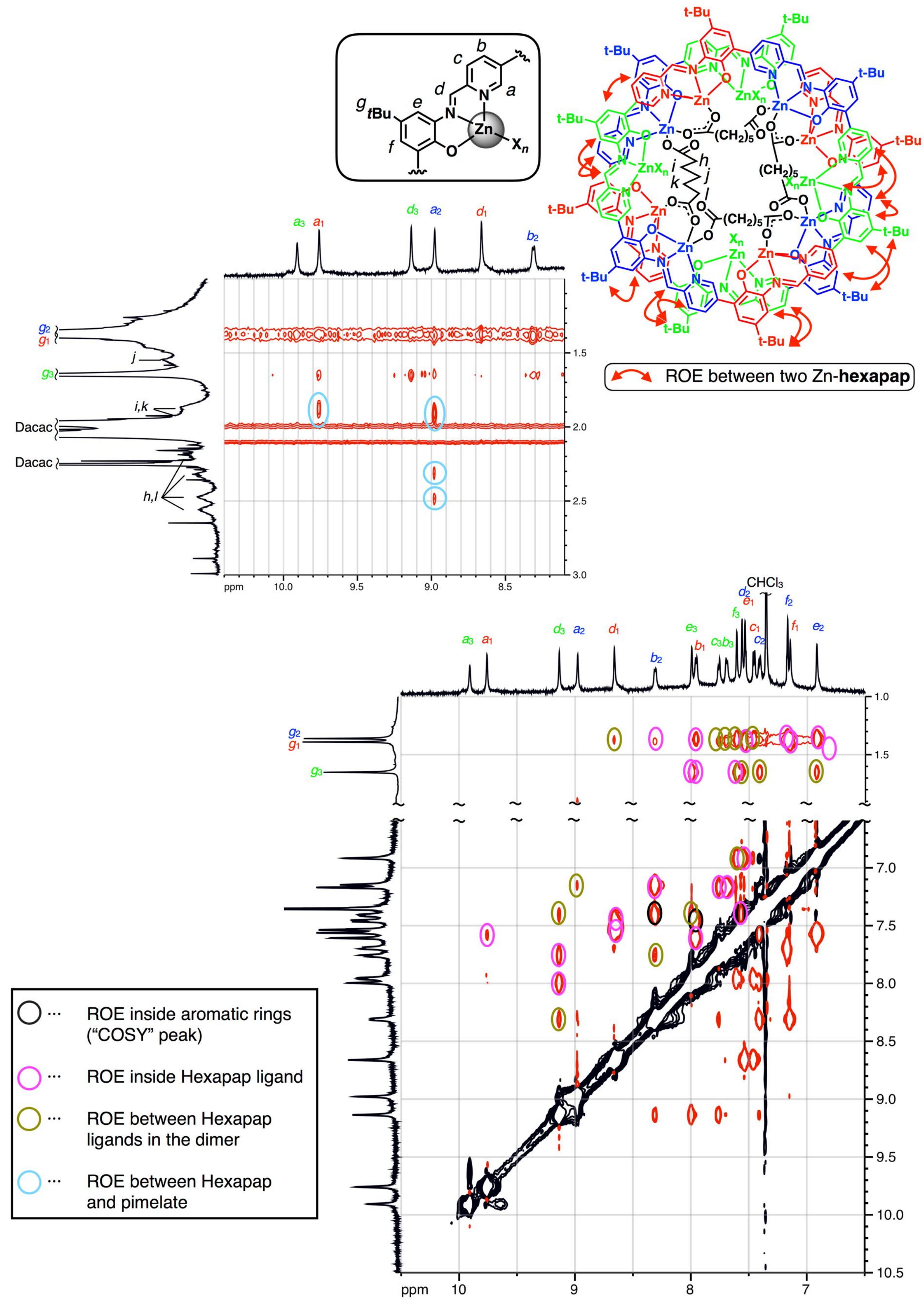

Supplementary Figure 22. ^{1}H–^{1}H ROESY NMR spectrum of [$\mathbf{1}_2Zn_{12}\mathbf{4e}_4X_n$] (X = labile coordinating ligand) (600 MHz, $CDCl_3/CD_3OD$ = 10:1, 298 K). Negative values are depicted in red in the spectrum.

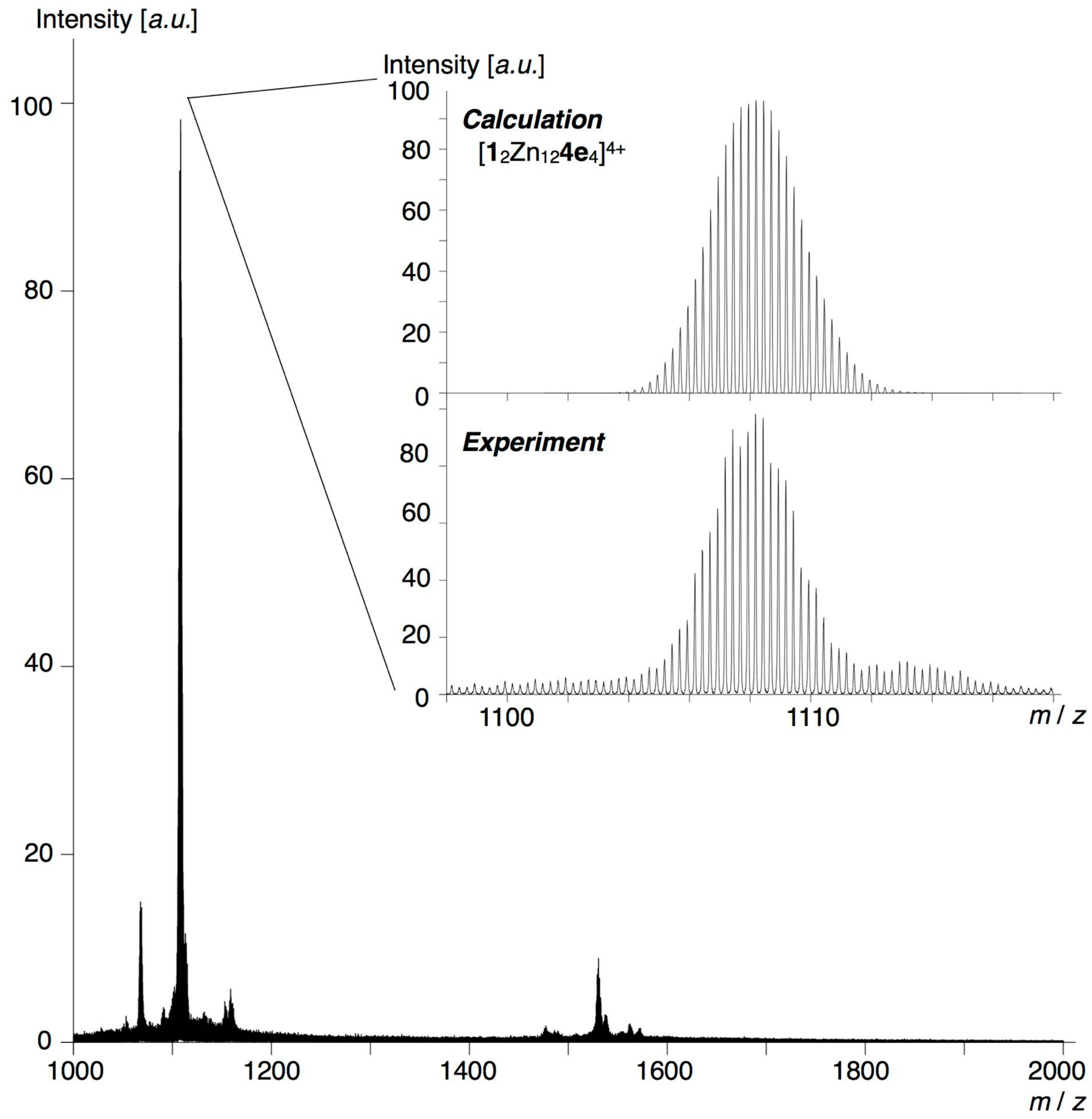

Supplementary Figure 23. ESI TOF mass spectrum of [$\mathbf{1}_2Zn_{12}\mathbf{4e}_4X_n$] (Solvent: CH_3OH, positive).

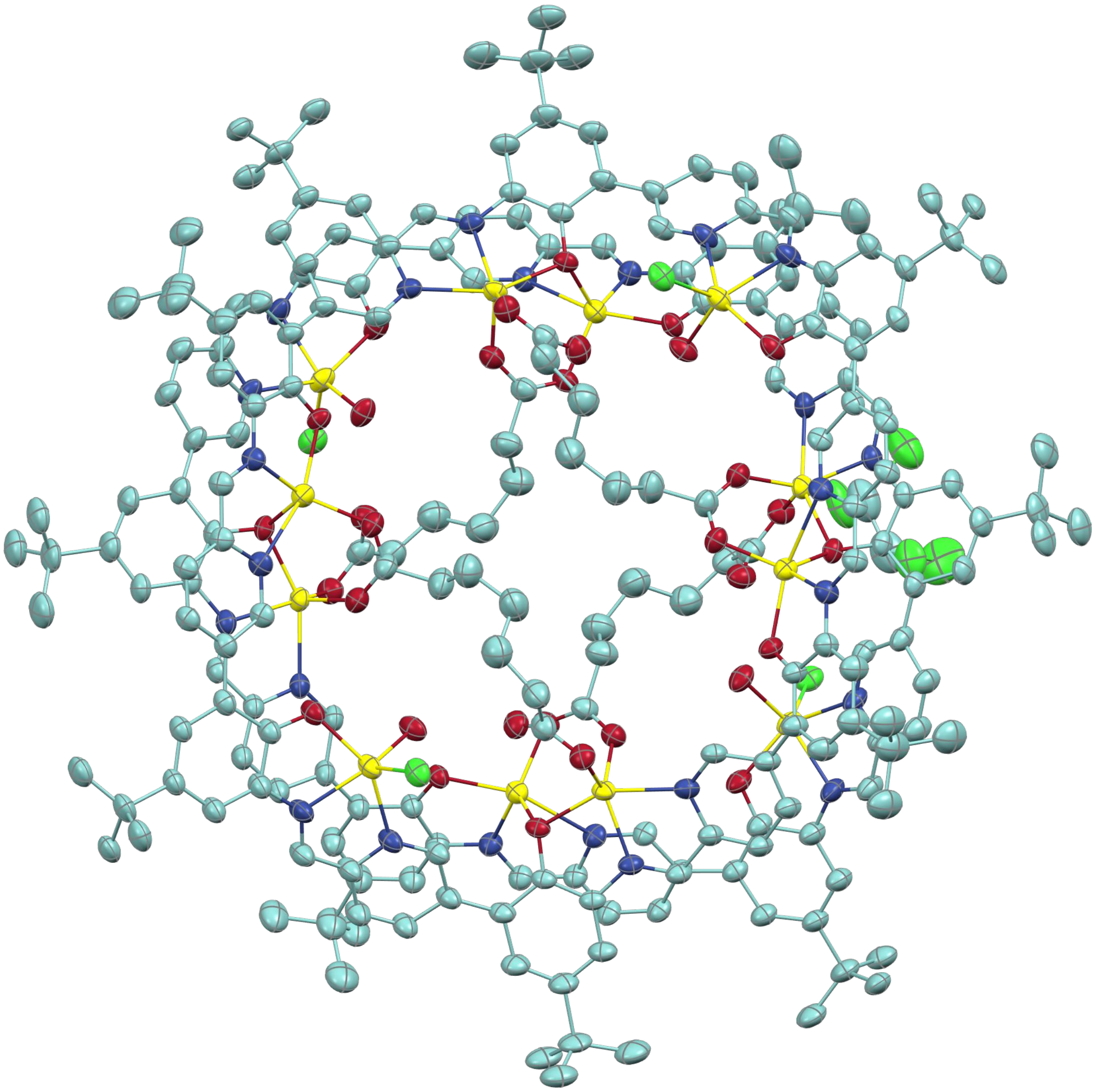

Supplementary Figure 24. The molecular structure of [$\mathbf{1}_2Zn_{12}\mathbf{4e}_4(H_2O)_4Cl_4$]·$0.5C_2H_2Cl_4$ determined by X-ray diffraction analysis. An ellipsoidal model (30% probability). Hydrogen atoms were omitted for clarity. One of the disorder patterns of *t*Bu groups is shown. C, light green; N, blue; O, red; B, yellow; Cl, green.

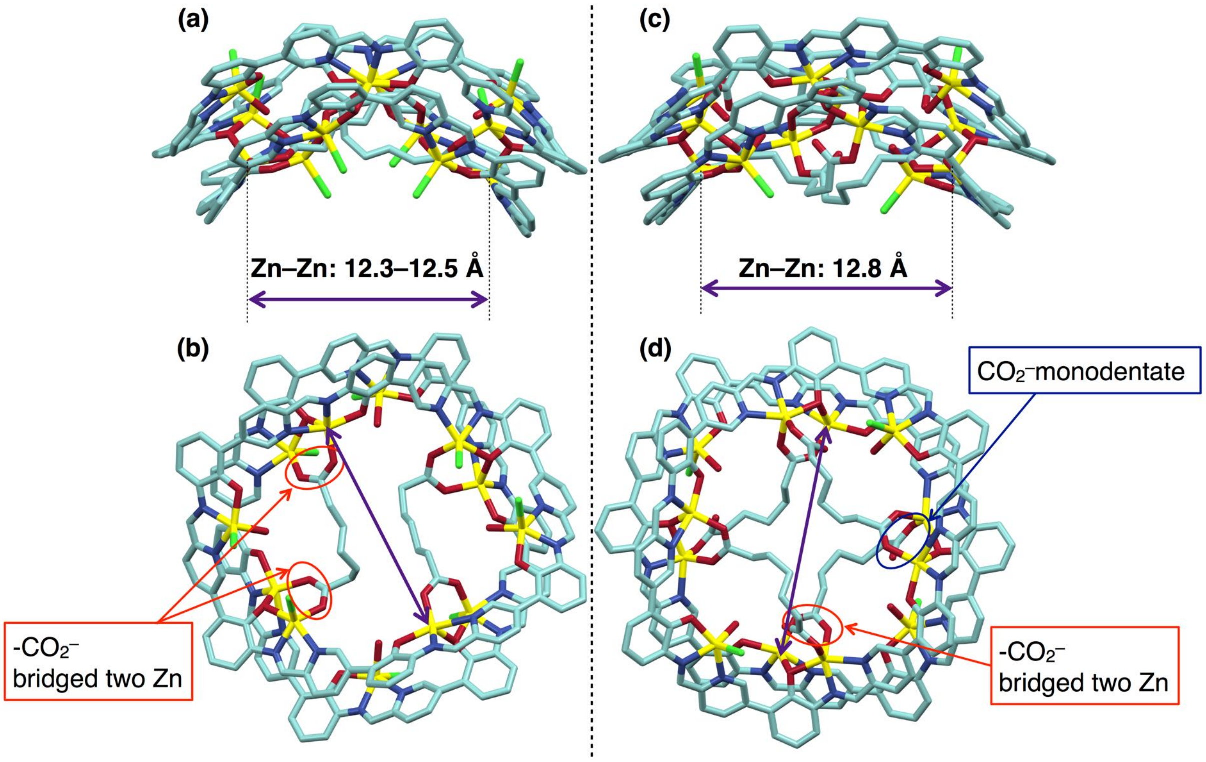

Supplementary Figure 25. Comparison of the molecular structures of the host-guest complexes. (a,b) [$\mathbf{1}_2Zn_{12}\mathbf{4e}_2(H_2O)_4Cl_8$]. (c,d) [$\mathbf{1}_2Zn_{12}\mathbf{4e}_4(H_2O)_4Cl_4$]. Diagonal distances between two Zn (part 1) atoms and between two Zn (part 4) atoms are shown in the figure. A stick model. Solvents, hydrogens, and *t*Bu groups were omitted for clarity. C, light green; N, blue; O, red; B, yellow; Cl, green.

Supplementary Table 1. Absorption and emission data of the Zn-hexapap [**1**$Zn_6(acac)_2$] upon binding of a series of dicarboxylic acids H_2**4a**–H_2**4i**.

Guest	λ_{abs} [nm]	λ_{em} [nm]	Φ_{em} [%]
No guest	546	762	1.7
Malonic acid H_2**4a**	546	705	0.5
Succinic acid H_2**4b**	546	720	1.2
Glutaric acid H_2**4c**	543	703	1.0
Adipic acid H_2**4d**	546	698	1.4
Pimelic acid H_2**4e**	529	698	1.9
Suberic acid H_2**4f**	544	705	1.1
Azelaic acid H_2**4g**	544	705	0.9
Sebacic acid H_2**4h**	550	710	0.6
Dodecanedioic acid H_2**4i**	554	710	0.7

Supplementary References

1. Nakayama, T. Composition and method for controlling arthropod pests, WO 2011049221, Apr 28, 2011.
2. Ojida, A., Sakamoto, T., Inoue, M., Fujishima, S., Lippens, G. & Hamachi, I. Fluorescent BODIPY-based Zn(II) complex as a molecular probe for selective detection of neurofibrillary tangles in the brains of Alzheimer's disease patients. *J. Am. Chem. Soc.* **131**, 6543–6548 (2009).
